# Duration of protective immunity and antibody responses in cattle immunised against alcelaphine herpesvirus-1-induced malignant catarrhal fever

**DOI:** 10.1186/1297-9716-43-51

**Published:** 2012-06-11

**Authors:** George C Russell, Julio Benavides, Dawn Grant, Helen Todd, David Deane, Ann Percival, Jackie Thomson, Maira Connelly, David M Haig

**Affiliations:** 1 Moredun Research Institute, Pentlands Science Park, Bush Loan, Penicuik, EH26 0PZ, Scotland, UK; 2 Instituto de Ganaderia de Montaña (CSIC-ULE), 24346, Grulleros (Leon), Spain; 3 School of Veterinary Medicine and Science, Nottingham University, Sutton Bonington, LE12 5RD, United Kingdom

## Abstract

Protection of cattle from alcelaphine herpesvirus-1 (AlHV-1)-induced malignant catarrhal fever (MCF) has been described previously, using an attenuated virus vaccine in an unlicensed adjuvant. The vaccine was hypothesised to induce a protective barrier of virus-neutralising antibody in the oro-nasal region, supported by the observation of high titre neutralising antibodies in nasal secretions of protected animals. Here we describe further analysis of this vaccine strategy, studying the effectiveness of the vaccine formulated with a licensed adjuvant; the duration of immunity induced; and the virus-specific antibody responses in plasma and nasal secretions. The results presented here show that the attenuated AlHV-1 vaccine in a licensed adjuvant protected cattle from fatal intranasal challenge with pathogenic AlHV-1 at three or six months. In addition, animals protected from MCF had significantly higher initial anti-viral antibody titres than animals that succumbed to disease; and these antibody titres remained relatively stable after challenge, while titres in vaccinated animals with MCF increased significantly prior to the onset of clinical disease. These data support the view that a mucosal barrier of neutralising antibody blocks infection of vaccinated animals and suggests that the magnitude of the initial response may correlate with long-term protection. Interestingly, the high titre virus-neutralising antibody responses seen in animals that succumbed to MCF after vaccination were not protective.

## Introduction

Malignant catarrhal fever (MCF) is a fatal disease of cattle and other ungulates caused by gamma-herpesviruses of the genus *macavirus*, including alcelaphine herpesvirus 1 (AlHV-1) and ovine herpesvirus 2 (OvHV-2) [1,2]. These viruses infect their reservoir hosts (wildebeest for AlHV-1 and sheep for OvHV-2) efficiently and without apparent disease but cause lymphoproliferative disease that is generally fatal when they infect susceptible hosts such as cattle, deer and bison. MCF is found worldwide wherever susceptible hosts mix with reservoir species [1].

The AlHV-1 and OvHV-2 genomes have been completely sequenced, revealing that they are highly-related viruses [3,4]. Both viruses cause MCF with similar pathology in susceptible animals that include laboratory species such as hamster and rabbit [5-7]. In cattle, MCF is characterised by fever, generalised lymphadenopathy, nasal and ocular discharge, and erosions of mucosal epithelia throughout the gastrointestinal tract. Death can occur within a few days or weeks after the first clinical signs. Characteristic lesions of this disease include non-purulent vasculitis and interstitial infiltration of lymphoid cells in most tissues, which may be associated with the presence of ulcers at epithelial surfaces [8,9].

Despite the severity of the lesions associated with MCF, virus-infected cells in the tissues have been difficult to detect, suggesting they were of low frequency [10]. However, recent results in AlHV-1 and OvHV-2-infected cattle, bison and rabbits suggest that virus-infected cells in MCF are more frequent [11,12]. These infected cells may be the source of the large granular lymphocytes that can be cultured from the tissues of MCF-affected animals. These cell lines comprise > 90% infected cells that have unrestricted cytotoxic activity for a variety of target cells [13-16].

Current control measures for MCF rely on minimizing contact between carrier animals and susceptible livestock. For AlHV-1 MCF, in areas close to wildebeest migration routes in east Africa, this has the effect of driving pastoralist cattle to poorer grazing where the risks of other diseases such as trypanosomiasis or east coast fever are increased, with consequent poor productivity [17,18]. Thus, improved control measures for MCF, particularly an effective vaccine, would significantly improve the livelihoods of subsistence-level pastoralists. Similarly, OvHV-2 MCF is a problem in farmed bison, deer and susceptible cattle worldwide, and a vaccine is eagerly sought. Early attempts to immunise cattle using live or inactivated formulations of attenuated strains of AlHV-1 were either unsuccessful or gave equivocal results [19,20]. However, we have recently developed an immunisation strategy that uses prime and boost immunisations of attenuated AlHV-1 in adjuvant intramuscularly in the upper neck to stimulate a mucosal barrier of virus–neutralising antibody in the oro-nasal pharyngeal region [21]. This approach successfully protected cattle challenged intranasally with a fatal dose of virulent AlHV-1. This original study used Freunds’ adjuvant and a relatively early time point for challenge (10 weeks after priming immunisation, 6 weeks after booster immunisation) but demonstrated that reliable protection against AlHV-1 MCF could be achieved.

In the present study, we wished to determine: firstly, whether the licensed oil-and-water adjuvant Emulsigen would facilitate protective immunity to AlHV-1; secondly, the duration of immunity; and thirdly, whether the degree of protective immunity correlated with virus-specific antibody responses locally (nasal secretions) or systemically (blood plasma). In these immunisation experiments AlHV-1 was used as, unlike OvHV-2, cell-free attenuated and virulent virus could be obtained from tissue culture and used to immunise cattle and challenge them respectively [20].

## Materials and methods

### Viruses and adjuvant

The strains of AlHV-1 used for vaccination and challenge were as described previously [21]. Briefly, the virulent C500 strain virus was collected from cultures of bovine turbinate (BT) cells infected with a cell suspension derived from pooled lymphoid tissue from rabbits infected with AlHV-1 C500 that had developed MCF. Infected BT cell cultures were passaged onto fresh BT cells by a 1:4 split four times at peak cytopathic effect (approximately weekly) after which virulent virus was harvested from culture supernatants and cells following three rounds of freeze-thaw treatment. Cell-free virus supernatant was stored at -80°C in batches and representative aliquots of each batch were titrated to allow calculation of the appropriate challenge dose. Titration measured 50% tissue-culture-infectious dose (TCID_50_) as described previously [21,22]. Pathogenic virus challenge in this experiment was by intranasal inoculation of 10 ml of virus suspension with titre approximately 10^4^ TCID_50_/mL.

The attenuated AlHV-1 C500 strain at passage > 1000 was used as the source of virus for immunisation [23]. This cell-free virus was obtained from BT cell culture supernatants, clarified by centrifugation and stored in batches at -80 °C. Representative aliquots of attenuated AlHV-1 were titrated as described for virulent AlHV-1.

Emulsigen is an oil-in-water adjuvant that has no ingredients of animal origin [24]. It contains micron-sized oil droplets with a high surface area available for antigen coating and does not cause adverse reactions at the injection site. Titration of the attenuated AlHV-1 virus in combination with adjuvant showed that 20% Emulsigen did not reduce the titre of the virus and that the adjuvant was not toxic to BT cells (data not shown). Thus, the AlHV-1-Emulsigen combination can be classed as an adjuvanted live vaccine.

### Animals

Disease-free and MCF seronegative male Ayrshire, Holstein or Friesian-Holstein cross calves of approximately 7 months of age were used in the experiments. The animal experiments were carried out with the approval of the Moredun Research Institute’s experiments and ethics committee and complied fully with the Home Office of Great Britain and Northern Ireland “Animals (Scientific Procedures) Act 1986”. Animals were assigned to experimental groups randomly, after taking age and breed into account. After virus challenge, animal rectal temperatures were measured daily and clinical scoring was done daily after the onset of fever (temperature > 40°C). Clinical scoring, based on temperature, body condition, ocular and nasal pathology, was used to ensure that all animals were euthanized at the onset of moderate clinical signs with an overdose of intravenous sodium pentabarbitone.

### Immunisation study design

Two vaccine studies are presented here. In the first study, the efficacy of the licensed adjuvant Emulsigen (MVP, Omaha, USA) was addressed (Table 1; group 1). Eight calves in this group were intranasally challenged with pathogenic AlHV-1 after prime and boost intramuscular (IM, upper neck) immunisation with attenuated AlHV-1 in Emulsigen (20% v/v), while the remaining eight calves were challenged after mock prime and boost immunisation with virus-free culture medium in Emulsigen.

**Table 1:** Immunisation study groups, treatments and outcomes

**Animal No.**	**Group**	**Vaccine/ Control**^a^	**Challenge day**^b^	**Day euthanized**^c^	**Virus DNA in blood at PM**	**Pathology**^**d**^**summary**	**Days to clinical MCF**
805	1	Vaccine	63	100	pos	MCF	37
789	1	Vaccine	63	103	pos	MCF	40
792	1	Vaccine	63	119	pos	MCF	56
568	1	Vaccine	63	139	neg	-	
570	1	Vaccine	63	139	neg	-	
785	1	Vaccine	63	140	neg	-	
822	1	Vaccine	63	140	neg	-	
807	1	Vaccine	63	140	neg	-	
575	1	Control	63	84	pos	MCF	21
787	1	Control	63	93	pos	MCF	30
814	1	Control	63	96	pos	MCF	33
808	1	Control	63	103	pos	MCF	40
785	1	Control	63	103	pos	MCF	40
149	1	Control	63	107	pos	MCF	44
821	1	Control	63	116	pos	MCF	53
781	1	Control	63	128	pos	MCF	65
861	2	Vaccine	77	152	neg	-	
702	2	Vaccine	77	152	neg	-	
214	2	Vaccine	77	153	neg	-	
774	2	Vaccine	77	153	neg	-	
769	2	Vaccine	77	153	neg	-	
090	2	Vaccine	77	154	neg	-	
967	2	Vaccine	77	154	neg	-	
968	2	Vaccine	77	154	neg	-	
165	2	Control	77	110	pos	MCF	33
700	2	Control	77	152	neg	-	
856	3	Vaccine	182	212	pos	MCF	30
776	3	Vaccine	182	217	pos	MCF	35
965	3	Vaccine	182	224	pos	MCF	42
701	3	Vaccine	182	257	pos	MCF	75
862	3	Vaccine	182	257	neg	non-specific	
611	3	Vaccine	182	257	neg	non-specific	
770	3	Vaccine	182	258	neg	-	
699	3	Vaccine	182	258	neg	-	
326	3	Control	182	217	pos	MCF	35
023	3	Control	182	250	pos	MCF	68
019	4	Vaccine	273	305	pos	MCF	32
969	4	Vaccine	273	310	pos	MCF	37
613	4	Vaccine	273	315	pos	MCF	42
771	4	Vaccine	273	320	pos	MCF	47
195	4	Vaccine	273	348	pos	MCF	75
966	4	Vaccine	273	348	neg	non-specific	
768	4	Vaccine	273	349	neg	-	
772	4	Vaccine	273	349	neg	-	
719	4	Control	273	300	pos	MCF	27
620	4	Control	273	302	pos	MCF	29

^a^ Vaccinated animals in all groups received the same dose (10 mL of 10^7^ TCID_50_ per mL) of vaccine for both prime and boost, given intramuscularly with 20% (v/v) Emulsigen. Control animals in each group received virus-free culture medium with Emulsigen, prepared and administered in the same way.

^b^ Pathogenic AlHV-1 challenge for each group was as follows: group 1 received 10 mL of 10^4.7^ TCID_50_/mL virus; group 2 received 10 mL of 10^3.8^ TCID_50_/mL virus; and groups 3 and 4 received 10 mL of 10^4.05^ TCID_50_/mL virus, administered intranasally in all groups.

^c^ All animals were monitored daily for clinical signs of MCF, including fever, loss of appetite, depression, nasal or ocular discharge and conjunctivitis. A clinical scoring scheme (Additional file 1 Table S 1) was used to ensure that all animals were euthanized at the onset of moderate clinical signs. Animal that did not develop clinical signs of MCF were euthanized for post-mortem at least 75 days after challenge. ^d^ Pathology was assessed as described in the text and is summarised as MCF, non-specific or negative (−). Tissue sections from animals with non-specific pathology were tested for presence of AlHV-1 DNA by real-time PCR. No viral DNA was detected in any sample with non-specific pathology.

In the second study, the longevity of protection induced by the vaccine formulated with Emulsigen was tested by immunisation of three groups of eight calves (Table 1; groups 2–4) followed by challenge at various time points after immunisation. In each experiment, uncoagulated blood and nasal secretions were collected from all animals just prior to primary immunisation, fortnightly until challenge and weekly thereafter as described previously [21].

At autopsy, the following tissues were collected: brain, buccal mucosa, rumen, reticulum, liver, kidney, lung, prescapular lymph node, mesenteric lymph node (MLN), naso-pharyngeal tonsil and blood. Pieces of each tissue (except blood) were fixed in 10% formal saline before processing and embedding in paraffin wax. For pathological analysis, 4 μm sections of formalin-fixed tissues were cut and stained with haematoxylin and eosin. In addition, total DNA was prepared from blood buffy coat cells or from sections of selected fixed tissues for PCR detection of viral DNA.

### Detection of viral DNA in blood and fixed tissues

Viral DNA was assayed in pure genomic DNA samples extracted from blood buffy coat cells or from 100 μm sections of fixed tissues by quantitative (q)PCR as described previously [25]. Briefly, ~50 ng of total DNA was assayed simultaneously for the presence of AlHV-1 and genomic actin sequences by duplex real-time PCR analysis. Each 20 μL assay contained 900 μM of each AlHV-1 forward and reverse primer (AlHV1-F, AlHV1-R) and 250 μM of the fluorogenic probe FAM-AlHV1 [25]; 450 μM of each genomic actin primer (gACT-F: 5′-CAC CTT CCA GCA GAT GTG GA-3′; gACT-R: 5′-CTA GAA GCA TTT GCG GTG GAC ) and 125 μM of the fluorogenic minor-groove binding (mgb) probe VIC-gACT (5′-VIC-AGC AAG CAG GAG TAC G-mgb) (K. Willoughby; unpublished data); and real-time PCR reagents containing Platinum Taq polymerase and ROX control dye (Invitrogen). All assays were conducted using standard conditions on ABI 7000 or 7500 sequence detection systems (Applied Biosystems).

### Analysis of antibody responses by ELISA and virus-neutralising antibody test

To quantify the humoral antibody response in blood plasma and the mucosal antibody response in nasal secretions (NS), an antibody ELISA was developed based on a detergent extract of the vaccine virus. Virus from cell-free culture fluid of BT cells infected with attenuated AlHV-1 C500 was concentrated by centrifugation (35 000 × *g*, 3 h, 4°C). Virus pellets were resuspended in phosphate-buffered saline (PBS) containing 0.8% CHAPS (3-[(3-Cholamidopropyl) dimethylammonio]-1-propanesulfonate, Sigma) and the insoluble virus pellet was collected by centrifugation as above. The virus pellet was then solubilised in 0.2% SDS before dialysing back into 0.2% CHAPS in PBS. This virus-positive ELISA coating antigen had a protein concentration of approximately 2 mg/mL. To produce a negative antigen for comparison, cell-free culture fluid from uninfected BT cells was treated according to the same protocol and the resulting solution used as negative ELISA antigen.

Pairs of adjacent rows of 96-well microtitre plates (Greiner, high protein binding) were coated with 50 μL of 5 μg/mL virus-positive or -negative antigen in 0.1 M carbonate buffer pH 9.6. Individual samples of blood plasma or sterile-filtered nasal secretion fluid diluted in PBS were then applied in duplicate to positive and negative antigen wells at serial dilutions from 1:100 to 1:4000. A standard curve, comprising 1:100 to 1:4000 dilutions of an AlHV-1-positive plasma pool was included on each plate to ensure reproducibility in the assays. A negative serum at 1:500 dilution was also included with each test sample series. Antibody bound in each well was detected using 1:1000 rabbit anti-bovine IgG-Horseradish Peroxidase conjugate (Sigma).

ELISA values (difference between means of positive and negative antigen wells for each sample dilution) were used to calculate a relative titre for each test sample, based on the standard curve produced from the positive control serum wells on the same plate. Test samples with ELISA values outside the range of the standard curve were discarded.

The virus neutralisation test was based upon inhibition of AlHV-1-induced cytopathic effect in BT cells by dilutions of plasma or nasal secretion fluid as described [21,22]. Assays were carried out in 96 well tissue culture plates with BT cells at greater than 80% confluence. All assays used a high titre bovine anti-AlHV-1 serum as a standard and included non-specific toxicity control wells containing sample and cells without virus.

### Pathology and histopathology

MCF was confirmed by clinical and pathological signs. The clinical symptoms included fever, nasal and ocular discharge, conjunctivitis and development of corneal opacity. Clinical assessment was done using a system (Additional file 1 Table S 1) which combined scores for pyrexia (1–3), nasal discharge (0–2), ocular pathology (1–3) and diarrhoea (1–6). Any animal with a score over 6 was euthanized without delay. This system ensured that all animals with clinical MCF were euthanized at a similar stage of the disease, prior to the development of moderate clinical signs. All animals with MCF had clinical signs for fewer than 5 days before euthanasia. Surviving animals were euthanized 11 weeks after pathogenic virus challenge. MCF histopathology in brain, kidney, liver, lung and digestive epithelium was examined and scored for the frequency of lesions consistent with MCF as described [21]. The pathology of each case was summarised as *MCF* (most of the organs studied contained significant numbers of lesions consistent with a diagnosis of MCF); *non-specific* (a small number of lesions, present usually only in one or two studied organs, and of mild intensity, without extensive infiltration of lymphocytes or clear vasculitis); or *negative* (no significant lesions observed). A positive test for AlHV-1 DNA in the blood on the day of post-mortem, in combination with MCF histopathological signs, was taken as a definitive diagnosis of MCF. In cases with non-specific pathology observed in some tissues, DNA prepared from adjacent sections was tested for the presence of virus DNA to assess whether the lesions observed could be due to AlHV-1 infection.

### Statistical analysis

Data for post-challenge survival between groups were analysed by Fisher’s exact test [26,27] while comparison of immune response data was done by students’ *T*-test on log(base2)-transformed titre data. *p* values less than 0.05 were considered significant.

## Results

### Protection of cattle from MCF by attenuated AlHV-1 in emulsigen

In these studies, four groups of eight cattle were vaccinated with attenuated AlHV-1 C500 in the presence of 20% (v/v) Emulsigen (MVP Labs, USA), while 14 control animals were mock-vaccinated with adjuvant in tissue culture fluid (Table 1). Both primary and boost immunisations, using the same material given four weeks apart, were administered by the intramuscular route, high in the neck of each animal.

Within groups 1 and 2 (Table 1) a total of 16 vaccinated animals were challenged with pathogenic AlHV-1 by the intranasal route at either 9 or 11 weeks after primary immunisation. Groups 3 and 4, each comprising eight vaccinated animals and two unvaccinated controls, were challenged at 26 and 39 weeks, respectively, after the primary immunisation, by intranasal inoculation of pathogenic AlHV-1 (Table 1).

Animal rectal temperatures and general demeanour (appetite, activity, nasal secretion) were recorded daily following challenge and additional clinical scores (Additional file 1 Table S 1) were recorded daily after the onset of fever (temperature ≥ 40°C). A final blood sample was collected before euthanasia and a range of tissues were collected post-mortem for diagnostic, pathological and molecular investigation. Animals that did not succumb to MCF were euthanized for post-mortem examination between 75 and 77 days after challenge (Table 1).

In groups 1 and 2, challenged at 9 or 11 weeks post vaccination, 13 animals were protected from clinical MCF while all but one of the control animals succumbed to disease. Analysis of these data by Fisher’s exact test showed that protection was statistically significant in each individual study (group 1, *p* = 0.025; and group 2, *p* = 0.003), while the combined results were highly significant (*p* < 0.0001), demonstrating the efficacy of Emulsigen as an adjuvant in this system.

In the groups challenged at later times after primary immunisation, four of eight animals in group 3, challenged at 26 weeks, and three of eight animals in group 4, challenged at 39 weeks, survived with no disease and no detectable AlHV-1 DNA post-mortem (Table 1). Comparison of the occurrence of MCF in the vaccinated and unvaccinated groups by Fisher’s exact test showed significant protection was afforded by the vaccine against MCF challenge at six months after immunisation (*p* = 0.04) but not at 9 months (*p* = 0.12).

### Evidence of circulating viral DNA in infected animals

Analysis of terminal blood and tissue samples showed that all animals with clear clinical and histopathological signs of MCF (Table 1) had detectable virus DNA in blood. In contrast, animals that showed no clinical disease or non-specific histopathological signs, as defined above (Table 1), had no detectable AlHV-1 DNA in blood or in tissue sections containing lesions, confirming that these animals did not have MCF at the time of euthanasia. In MCF cases in either vaccinated or control animals, virus DNA could be detected up to three weeks before post mortem.

### ELISA analysis of antibody responses following vaccination and challenge

The relative titres of AlHV-1-specific antibodies in nasal secretions and blood plasma were determined with respect to standard pools of MCF-positive plasma or nasal secretions using the direct ELISA described above. Animals in group 1 were assayed independently of the other groups, using a different serum pool to calculate relative titre. Thus the ELISA results from group 1 are not comparable with the other groups. For animals in groups 2–4, samples from throughout the study period were tested together. Unvaccinated control animals challenged with AlHV-1 showed no detectable titre of anti-viral antibodies at any point before or during the progress of clinical disease.

**Figure 1:**
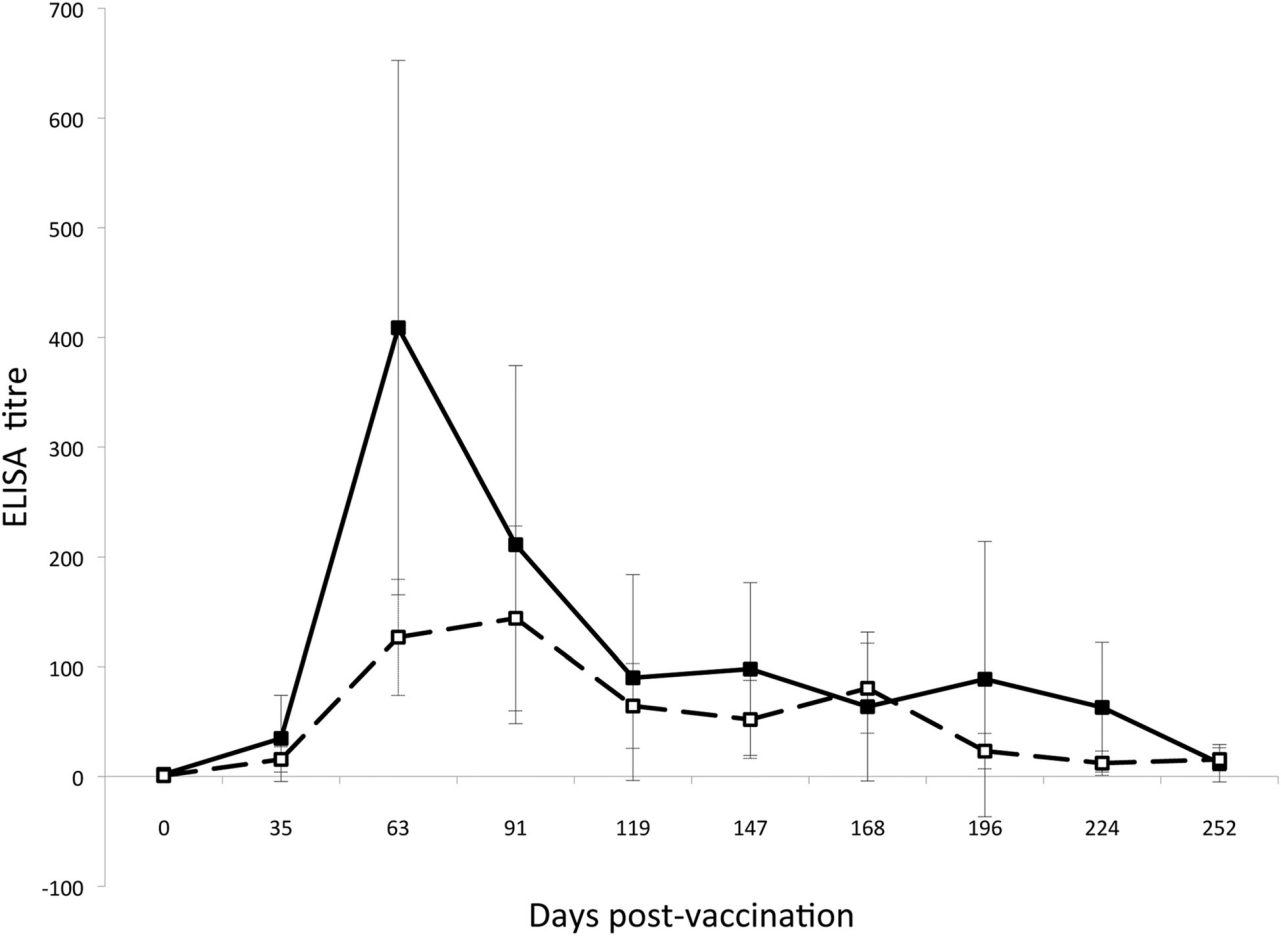
**ELISA analysis of virus-specific antibody responses in vaccinated animals from groups 2–4 prior to challenge.** Average virus-specific antibody titres in plasma (filled squares, solid line) and in nasal secretions (open squares, broken line) were plotted for all unchallenged animals available at each time point. Error bars correspond to the standard error of the means.

**Figure 2:**
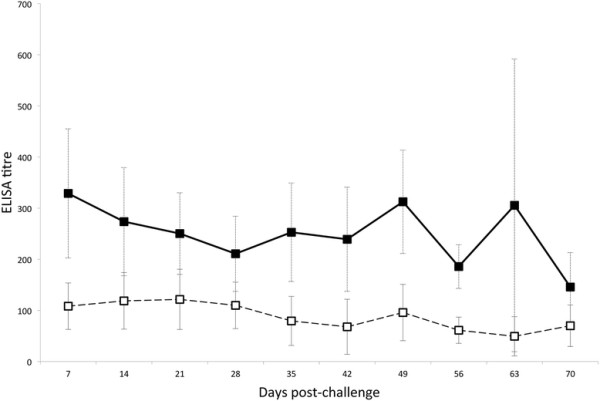
**ELISA analysis of virus-specific antibodies in vaccinated animals from groups 2–4 that were protected from MCF after challenge.** Average virus-specific antibody titres in plasma (filled squares, solid line) and in nasal secretions (open squares, broken line) are plotted for all vaccinated and challenged animals that survived to the end of the trial without clinical MCF. Error bars correspond to the standard error of the means.

**Figure 3:**
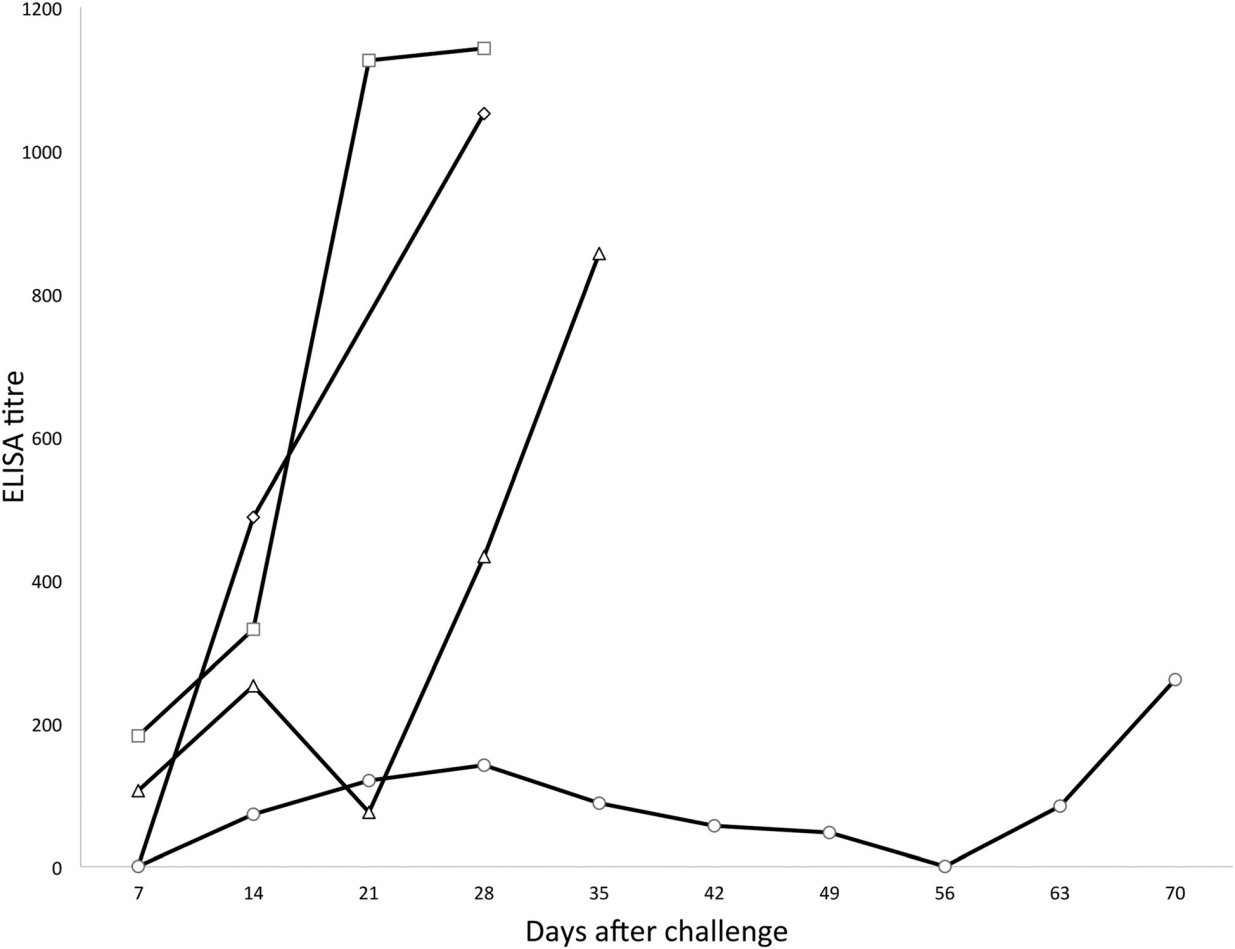
**ELISA analysis of virus-specific antibodies in the four animals from group 3 that succumbed to MCF after challenge.** Relative titres of antibodies in the nasal secretions of each animal at a range of timepoints after challenge are plotted individually. Each animal is represented by a different symbol, joined by solid lines. The rightmost point in each line corresponds to the sample collected on the day of post mortem.

Following immunisation and boost, all vaccinated animals showed a peak in anti-virus antibody titre in both blood plasma and nasal secretions between days 63 and 91 after primary immunisation (Figure 1). Average titres then reduced to less than 1/100 in plasma and nasal secretions by day 120. Analysis of virus-specific antibody titres in nasal secretions and plasma at a point within the peak response to vaccination (day 63), showed that plasma antibody titres were significantly lower on day 63 in animals that went on to develop MCF than in those that were protected (*p* = 0.006) while nasal secretion antibody titres were not significantly different between these groups. After challenge, the patterns of antibody response also differed between vaccinated animals that succumbed to MCF and those which did not (Figures 2 and 3). Animals protected by vaccine showed a generally stable virus-specific antibody response (Figure 2) with average titres in both plasma and nasal secretions remaining below about 1/300. In contrast, the animals that succumbed to MCF after vaccination showed an increase in anti-viral antibody titre that preceded the development of clinical MCF. This is illustrated in Figure 3 for the nasal secretion antibody responses of the four animals in group 4 which succumbed to MCF. It is notable that the increase in antibody titre is not related to the timing of challenge but to the onset of MCF in each animal (Figure 3). Analysis of groups 3 and 4 showed that vaccinated animals with MCF had significantly higher anti-viral antibody titres in nasal secretions at the time of euthanasia (*p* = 0.03) than animals that were protected. A similar trend was observed in plasma but this was not significant (*p* = 0.07).

### Virus-neutralising antibody responses

**Figure 4:**
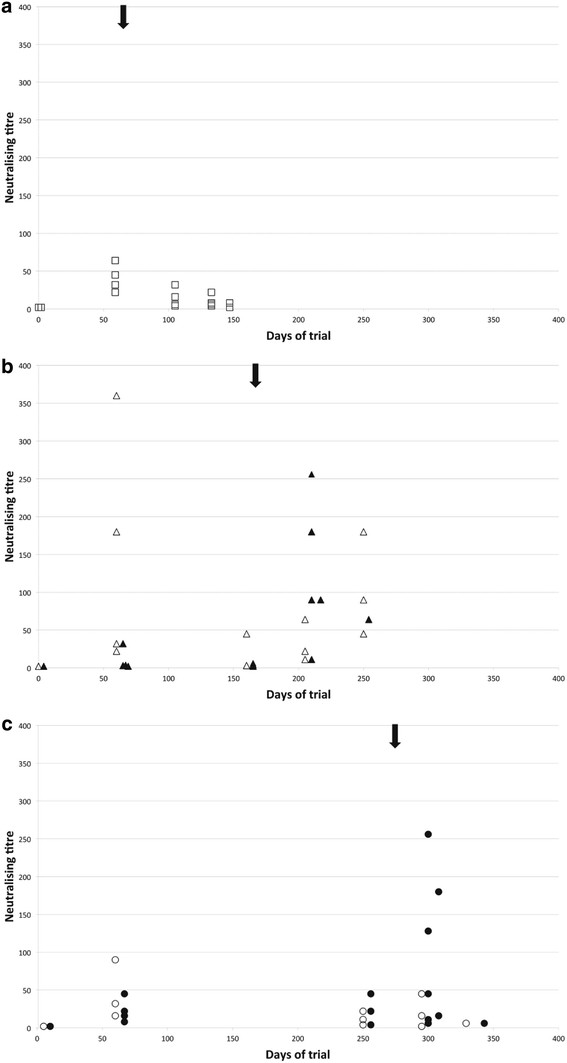
**Virus neutralising titres observed in nasal secretion samples from vaccinated animals in groups 2–4 at a range of time-points.** The arrow above each graph indicates the timing of challenge for that group. Samples from animals in group 2 (panel A) are represented by squares, group 3 (panel B) by triangles and group 4 (panel C) by circles. Filled symbols represent animals that succumbed to MCF while open symbols represent animals protected by vaccination.

Virus-neutralising antibody titres (VNT) were assayed in samples from all animals at a selection of time points and for all terminal samples. Group 2 animals (none of which succumbed to MCF) did not show a significant increase in VNT following challenge (Figure 4a); while animals in groups 3 and 4 that succumbed to MCF had increased VNT at timepoints after challenge compared to animals that were protected (Figure 4b, 4c) Thus, neutralising antibody titres roughly paralleled ELISA titres and confirmed the observation from ELISA data that animals which succumbed to MCF after vaccination (in groups 3 and 4) had lower titres at day 63 (*p* = 0.03 plasma, *p* = 0.01 nasal secretions) and higher titres in terminal plasma (*p* = 0.005) than animals from the same groups that were protected.

## Discussion

The data presented in this paper extend previous work showing that a vaccine based on attenuated AlHV-1 protected cattle challenged intranasally with pathogenic AlHV-1 C500 from developing clinical MCF [21]. This previous study reported that protection could be obtained by immunisation with vaccine formulated with Freunds adjuvant, an unlicensed product. We have also demonstrated that the attenuated virus without adjuvant did not protect from intranasal challenge with pathogenic AlHV-1 (G.C. Russell, unpublished data). Here we show that the licensed oil-in-water adjuvant Emulsigen can facilitate protection from intranasal experimental AlHV-1 challenge given up to six months after primary immunisation.

Analysis of plasma and nasal secretion antibody responses to vaccination and challenge showed that (i) unvaccinated animals with MCF had no ELISA-detectable or neutralising antibody response to AlHV-1 following challenge; (ii) vaccinated animals that were protected from MCF had significantly higher titres of virus-neutralising antibodies in both plasma and nasal secretions at about 1 month after boost than unprotected animals and these titres did not significantly change after challenge (Figure 4); (iii) in contrast, vaccinated animals that succumbed to MCF showed significant increases in both total virus-specific antibody and virus-neutralising antibody during the development of clinical MCF (Figure 3 and Figure 4).

The lack of a detectable antibody response in unvaccinated cattle challenged with AlHV-1 could reflect the rapid onset of MCF that might prevent the induction of an immune response to combat the infection. However, in the experiments reported here, the appearance of clinical MCF in control animals ranged between 21 and 68 days after challenge (Table 1), suggesting that there was sufficient time for the development of an immune response in at least some of the infected cattle. These data confirm and extend the previous observation that control cattle challenged intranasally did not develop detectable titres of virus-neutralising antibody [21].

In field cases of MCF, virus-specific antibody responses are reported to be found in 70-80% of samples tested [22]. The lack of antibody response in some MCF cases was confirmed in a comparative analysis of MCF-specific PCR and serological diagnostic testing where 14 of 39 OvHV-2 PCR-positive animals with clinical MCF were considered serologically borderline or negative [28]. Additionally, sub-clinical infection with MCF viruses has been inferred from serological surveys in which animals from MCF-susceptible species (cattle, bison, deer) had detectable MCF-specific antibodies or viral DNA in the absence of disease [29-31]. More recently, experiments using OvHV-2 to infect cattle or bison showed sub-clinical infection of five cattle, with seroconversion, and of one bison without seroconversion; while evidence of previous infection was found in six bison of which two had both detectable anti-MCF antibodies and OvHV-2 DNA [32,33]. Previous subclinical infection did not appear to reduce susceptibility to MCF following intranasal challenge.

These observations suggest that infection with MCF viruses and the ensuing immune response and disease may be more complex than previously thought, leading to the development of MCF in some animals without a detectable virus-specific antibody response. There is a lack of good transmission and epidemiological work on MCF that requires to be done to address such issues.

In contrast to the boost to AlHV-1-specific antibody responses found in vaccinated animals that developed MCF, vaccine-protected animals did not show a significant increase in antibody titres after challenge. This is likely to be because no virus in immunogenic quantity was able to penetrate the mucosal barrier of neutralising antibody to boost the response. This supports the contention that the establishment of a mucosal barrier of antibody is a mechanism of protection against MCF. This is reinforced by the observation that protected animals in groups 3 and 4 had high virus-neutralising antibody titres after immunisation compared to animals that succumbed to MCF. The higher titres are likely to be associated with longer duration of antibody responses and hence protection at later time points.

The development of high titre virus-specific antibody responses prior to the onset of MCF among vaccinated cattle in these experiments suggests that the development of clinical MCF includes the expression of virus gene products that stimulate an anamnestic immune response. Indeed, clinical MCF cases are often characterised by the development of a circulating antibody response which includes antibodies specific for virus structural antigens including the major glycoprotein complex of the viral envelope [34,35]. This is in contrast to recent suggestions that MCF in rabbits and cattle is associated with a latent infection of lymphocytes and a lack of lytic gene expression that would include antigenic capsid and envelope proteins [11,36,37]. The presence of antibodies specific for virus structural proteins in MCF-affected animals suggests that infection results in a pattern of virus gene expression that includes a number of lytic cycle antigens but without the production of infectious virus. It may therefore be inappropriate to discuss MCF in terms of lytic or latent gene expression patterns since latency should be defined in the natural host rather than in MCF-susceptible species that cannot propagate these viruses.

Current work aims to improve the magnitude and duration of the protective immune response by the strategic inclusion of toll-like receptor (TLR) agonists in the MCF vaccine. In addition, the role, if any, of cytotoxic T cells in protection against MCF is currently not known and this is being investigated. The six month window of protection offered by the current immunisation regime should be adequate to protect cattle in Eastern and Southern Africa exposed to wildebeest during the calving period. This is being studied in field trials in Tanzania.

## Competing interests

The authors declare they have no competing interests

## Authors’ contributions

Experimental design and planning: GCR, DG, HT, JT, JB, DMH; animal experiments GCR, DG, HT, JT, JB; pathology analysis DG, HT, JB; serological analysis DD, AP, MC, DG; data processing and statistical analysis DD, AP, DG, DMH, GCR; drafting of the manuscript GCR, DMH, DD, JB. All authors read and approved the manuscript.

## Supplementary Material

Additional file 1 **Clinical scoring scheme for cattle with MCF.** Cattle were scored daily following the onset of fever > 40°C. Animals were euthanized when their daily score totalled more than 6. However, any animal with symptoms that compromised its welfare would be euthanized immediately. Click here for file
