# Supplementary material for: Duration of protective immunity and antibody responses in cattle immunised against alcelaphine herpesvirus-1-induced malignant catarrhal fever
Source: Vet Res. 2012 Jun 11;43(1):51. doi: 10.1186/1297-9716-43-51 (PMC3425131; doi:10.1186/1297-9716-43-51)
Supplement: Additional file 1 — Clinical scoring scheme for cattle with MCF. Cattle were scored daily following the onset of fever > 40°C. Animals were euthanized when their daily score totalled more than 6. However, any animal with symptoms that compromised its welfare would be euthanized immediately. [file 1297-9716-43-51-S1.doc]

**Supplementary Table S1 Clinical scoring scheme for cattle with MCF.**

**Cattle were scored daily following the onset of fever > 40 °C. Animals were euthanized when their daily score totalled more than 6. However, any animal with symptoms that compromised its welfare would be euthanized immediately.**

| **Condition** | **Comment** | **Score** |
| --- | --- | --- |
| Fever > 40.0 °C | maximum score of 3 | 1 per day |
|  |  |  |
| Diarrhoea | slight | 1 |
|  | moderate | 2 |
|  | haemorrhagic | 6 |
| Nasal discharge | clear mucous | 0 |
|  | opaque mucous | 1 |
|  | fibrinous mucous | 2 |
| Ocular pathology | clear discharge | 1 |
|  | Conjunctivitis /inflammation around eye/partial opacity | 2 |
|  | complete opacity | 3 |
